# Influence of Body Functions and Structures on Home Return Through Activity and Participation: A Mediation Analysis in Patients With Stroke

**DOI:** 10.7759/cureus.95111

**Published:** 2025-10-21

**Authors:** Daisuke Kimura, Hiroki Bizen, Kenta Kunoh, Daiki Nakashima, Terufumi Iituka

**Affiliations:** 1 Division of Occupational Therapy, Department of Rehabilitation, Faculty of Health Sciences, Naragakuen University, Nara, JPN; 2 Department of Occupational Therapy, Faculty of Health Sciences, Kansai University of Health Sciences, Osaka, JPN; 3 Graduate School of Health Sciences, Department of Health Sciences, Kansai University of Health Sciences, Osaka, JPN

**Keywords:** activities and participation, body function and structure, mediation analysis, sem, stroke

## Abstract

Objective: This study aimed to examine whether body function and structure directly influences discharge home or exerts an indirect effect via activity and participation in patients with stroke, employing mediation analysis.

Methods: Sixty-seven inpatients with stroke in a convalescent rehabilitation ward were retrospectively examined. Latent variables for body function and structure (grip strength, knee extension strength, six-minute walk test, Berg Balance Scale) and activity and participation (13 motor items from the Functional Independence Measure) were developed. Structural equation modeling assessed associations with home return.

Results: The direct effect of body function and structure on home return was not significant (β = 0.474, p = 0.0786). In contrast, the indirect effect via activity and participation was significant (β = -0.866, p = 0.001).

Conclusion: Enhancement of body function alone does not sufficiently promote home return in patients with stroke. Improving activity and participation is vital for effective discharge planning, underscoring the essential role of occupational therapy in rehabilitation.

## Introduction

Physical therapists (PTs) and occupational therapists (OTs) are central to rehabilitation; however, they differ in their areas of focus and intervention methods. In addition, rehabilitation physicians (physiatrists) play a critical integrative role in coordinating multidisciplinary rehabilitation care. They provide diagnostic synthesis, manage comorbidities such as spasticity, fatigue, and depression, and set individualized rehabilitation goals. By combining pharmacological and non-pharmacological interventions, they optimize neuroplasticity and guide the therapeutic process. Their involvement ensures that physical and mental function improvements are aligned with medical stability and overall recovery planning. Physical therapists primarily target improvements in “physical and mental functions and structures,” emphasizing walking ability in patients with neurological disorders and older adults [1-3]. They also implement interventions addressing various aspects of physical and mental functions, including exercise therapy and joint range of motion training to maintain and enhance balance and mobility, which are essential to walking function [4-6].

Conversely, occupational therapists prioritize supporting “activities” and “participation” to enhance quality of life (QOL) and well-being through a holistic approach [7-10]. OT interventions assist clients in fulfilling meaningful roles across diverse settings such as the home, school, workplace, and community, focusing not only on functional recovery but also on realizing “a life that is true to the individual” [7,10]. Occupational therapy employs a client-centered approach, respecting subjective values, including “what they want to do” and “what they need to do,” and adapts activities and environments accordingly [8,10-13].

Moreover, contemporary rehabilitation science emphasizes the biological foundations of recovery, such as neural plasticity, motor learning, and cortical reorganization. These mechanisms explain why targeted rehabilitation interventions, such as task-specific training and exercise therapy, promote functional network reorganization and improve walking and balance in neurological patients. Exercise and activity-based interventions are not merely mechanical; they trigger synaptic remodeling and upregulate neurotrophic factors, such as brain-derived neurotrophic factor (BDNF) and insulin-like growth factor-1 (IGF-1), which contribute to neural repair and adaptation. Incorporating these perspectives situates rehabilitation within a mechanism-based neurorehabilitation framework, linking “physical and mental functions” to measurable biological processes.

Rehabilitation practice thus encompasses two main strategies: one aims to reduce activity limitations and participation restrictions by improving physical and mental functions, thereby facilitating return to home living; the other directly targets impairments in activities and participation to support life reconstruction. The first approach considers “physical and mental functions and structures” as direct determinants of home return, whereas the second views “activities and participation” as mediators indirectly influencing home return. 

However, although many studies have shown that both body function and activities of daily living (ADL) independence are predictors of home discharge after stroke, the causal pathway, that is, whether improvements in physical function directly promote home return or do so indirectly through enhanced activity and participation, has not been fully established. Recent systematic reviews and mediation analyses indicate partial evidence of such pathways [13-15], but their mechanisms remain inconclusive. Therefore, further verification using mediation frameworks is warranted.

Previous research in stroke rehabilitation has also begun to apply mediation or structural equation modeling (SEM) to clarify causal pathways among body function, ADL performance, and discharge outcomes. For instance, Chen et al. [16] used SEM to identify functional factors influencing post-acute stroke outcomes, while Lin et al. [14] and Kenta et al. [15] explored indirect effects between physical function and home discharge through ADL performance. These studies highlight the value of modeling both direct and indirect relationships, but the mediating role of activity and participation remains insufficiently examined.

The purpose of this study is to clarify the causal structure-whether “physical and mental functions and structures” directly influence return to home life or indirectly influence it through “activity and participation”-using mediation analysis.

## Materials and methods

The study included 67 patients with stroke (41 males and 26 females, mean age 72.6 ± 12.3 years) hospitalized in a rehabilitation ward during the recovery phase. Patients transferred to an acute care hospital or who died during hospitalization were excluded.

Data collection was conducted retrospectively. Two research collaborators created the dataset based on electronic medical records. All evaluation data were obtained at discharge. To ensure data quality and minimize bias, all data extraction was independently conducted by two licensed occupational therapists trained in Functional Independence Measure (FIM) scoring and physical function assessment. The extracted data were cross-checked, and any discrepancies were resolved through discussion and consensus. Inter-rater reliability for FIM scoring was maintained through adherence to standardized assessment manuals and periodic calibration meetings. Regarding missing data, cases with incomplete outcome variables were excluded after confirming that the proportion of missing values was below 5%, thus minimizing potential bias from data omission.

Seven indicators assessed physical function: (1) grip strength (affected/non-affected side), (2) knee extension muscle strength to body weight ratio (affected/non-affected side), (3) six-minute walk test (6MWT), and (4) Berg Balance Scale (BBS). For activity and participation, the 13 motor items of the Functional Independence Measure (FIM) were used: eating, grooming, bathing, dressing upper, dressing lower, toileting, sphincter control (bladder/bowel), transfers (bed/chair/tub), and locomotion (walk/wheelchair, stairs). The FIM assesses independence on a seven-point scale from full independence to total dependence. Only motor items were analyzed. The Functional Independence Measure (FIM) is widely used in clinical settings in Japan, and its application for research purposes does not require additional permission. We confirm that no copyrighted materials were reproduced, and only the scoring data routinely collected in clinical practice were analyzed. The FIM was developed by the Uniform Data System for Medical Rehabilitation, a division of UB Foundation Activities Inc., which holds its copyright. Although no certificate of permission is issued in Japan, the FIM Version 3 is approved for research use within Japan. The FIM used in this study conforms to Version 3.

Continuous variables were expressed as mean ± standard deviation (SD), and categorical variables as N (%). Sex was presented separately as male and female in N (%). To examine whether activity and participation mediated the relationship between body function/structure and home discharge, a mediation analysis was performed. The analysis was conducted using Amos version 7.0 (IBM Corp., Armonk, NY, USA). Standardized path coefficients were calculated, and model fit was evaluated using indices including the goodness-of-fit index (GFI), adjusted goodness-of-fit index (AGFI), Tucker-Lewis index (TLI), comparative fit index (CFI), and root mean square error of approximation (RMSEA). Acceptable model fit thresholds were pre-specified based on conventional SEM criteria: GFI, AGFI, TLI, and CFI values ≥ 0.90 and RMSEA ≤ 0.08 were considered indicative of an acceptable fit. These criteria were used as reference points to evaluate the adequacy of the mediation model. A two-sided p-value of <0.05 was considered statistically significant.

Statistical analyses were performed using IBM SPSS Statistics version 28.0 and Amos 7.0 (IBM Corp., Armonk, NY, USA) with a significance level of 5%.

The theoretical framework of this study was based on the International Classification of Functioning, Disability and Health (ICF), in which “Body Function & Structure” represents physiological and anatomical integrity, “Activity & Participation” represents the execution of daily tasks and social involvement, and “Home return” reflects reintegration into the living environment. The indicators used for each construct were standardized and validated tools: grip strength, knee extension strength, 6MWT, and BBS for Body Function & Structure; the 13 motor items of the Functional Independence Measure (FIM) for Activity & Participation; and the discharge destination (home or not) for home return.

Mediation analysis using SEM was selected to test the indirect pathway between body function and home return via activity and participation, allowing simultaneous estimation of direct and indirect effects while accounting for measurement errors. Although path analysis could also test such relationships, SEM was chosen as it incorporates latent variables representing each construct.

Ethics statement

This study was conducted in accordance with the Declaration of Helsinki. Ethical approval was obtained from the Research Ethics Review Committee of Kansai University of Health Sciences (approval number: 22-18). Given the retrospective nature of the study, informed consent was obtained through an opt-out procedure, and patients were provided the opportunity to refuse participation.

## Results

**Figure 1:**
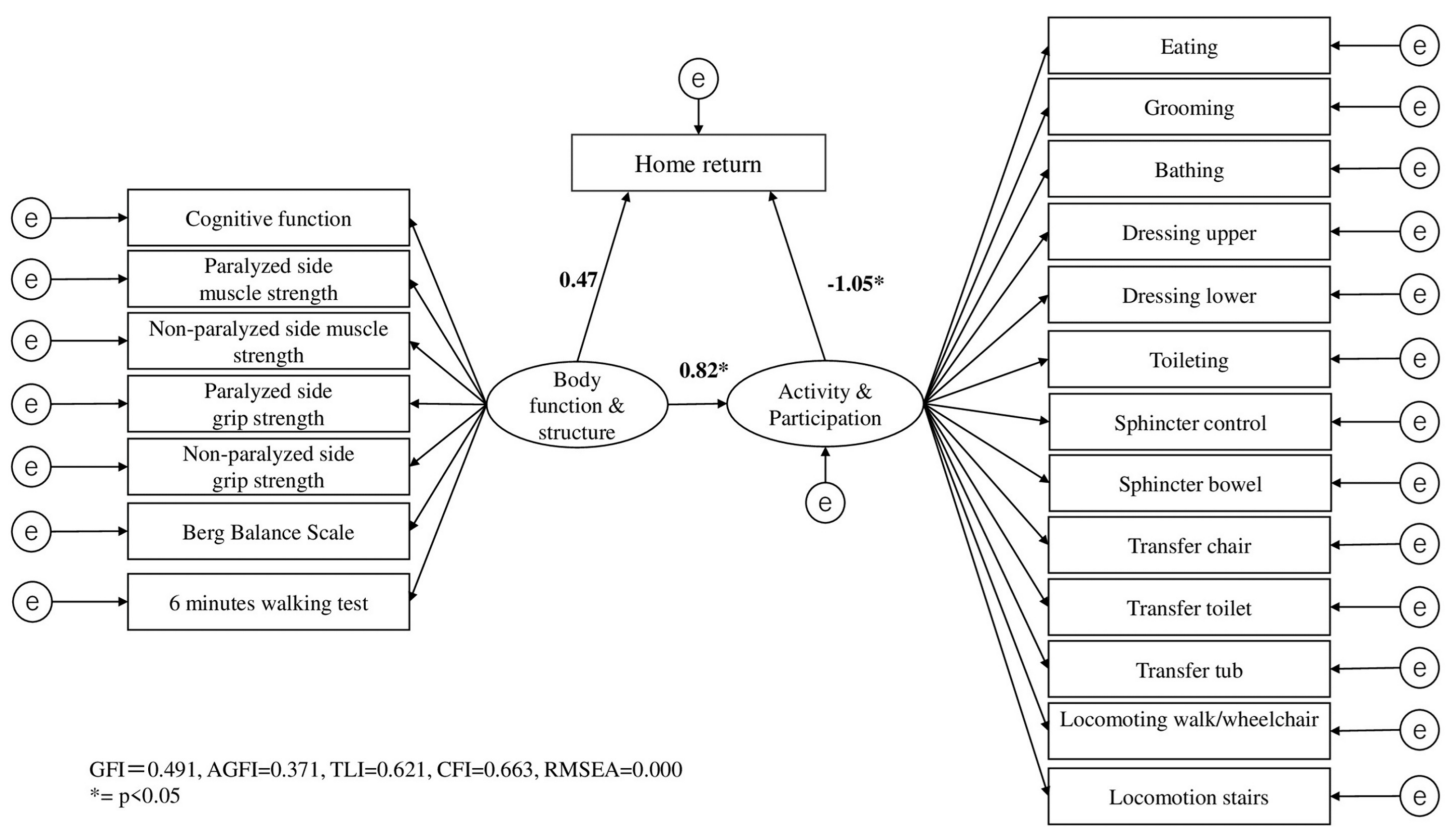
SEM results. SEM: structural equation modeling, GFI: goodness-of-fit index, AGFI: adjusted goodness-of-fit index, TLI: Tucker-Lewis index, CFI: comparative fit index, RMSEA: root mean square error of approximation.

In this study, mediation analysis evaluated the direct effect of “Body function and structure” on “Home return” and the indirect effect mediated by “Activity and participation.” The standardized coefficient for the direct effect was 0.474, which was not statistically significant (p = 0.0786, 95% CI: -0.001 to 0.007) (Table 1). Conversely, the standardized coefficient for the indirect effect through “Activity and participation” was -0.866, reaching statistical significance (p = 0.001, 95% CI: -0.008 to -0.001) (Table 1). Goodness-of-fit indices for the mediation model were GFI = 0.491, AGFI = 0.371, TLI = 0.621, CFI = 0.663, and RMSEA = 0.000 (Figure 1).

**Table 1:** Multiple mediation model: Indirect effects of SC on comp. Results of mediation analysis for the relationship between body function and structure, activity and participation, and home return. Values are standardized coefficients. p < 0.05 was considered statistically significant. *p < 0.05, **p < 0.01. SC: standardized coefficient.

	Products of coefficients				Bootstrapping bias-correlated 95% CI	
Relationship	B	b	SE	p	Lower	Upper
Body function and structure → Home return	0.002	0.474	0.001	0.086	-0.001	0.007
Body function and structure → Activity and participation → Home return	-0.003	-0.866	0.003	0.001**	-0.008	-0.001
Bootstrap sample size = 2000; SE = standard error; CI = confidence interval, **p < 0.01						

Descriptive statistics of the participants are presented in Table 2. The mean age of the participants was 72.6 ± 12.3 years, with 61.2% being male. The average grip strength on the affected side was 17.1 ± 17.5 Kg, while on the non-affected side it was 24.9 ± 13.1 Kg. The mean knee extension strength to body weight ratio was 29.5 ± 14.3 Nm/kg on the affected side and 40.5 ± 17.5 Nm/kg on the non-affected side. The average 6MWT distance was 273.9 ± 13.1 meters, and the mean BBS score was 42.1 ± 15.2. The average motor FIM score at discharge was 71.1 ± 20.1. These findings indicate that improvement in body function alone was insufficient for home discharge, whereas the pathway through activity and participation played a critical mediating role.

**Table 2:** Characteristics of participants. Values are presented as mean ± SD or N (%). FIM: Functional Independence Measure.

Variable	Value
Age (years), Mean ± SD	72.6 ± 12.3
Sex, N (%)	-
Male	41 (61.2%)
Female	26 (38.8%)
Grip strength affected side (Kg)	17.1 ± 17.5
Grip strength non-affected side (Kg)	24.9 ± 13.1
Knee extension strength/body weight affected side (Nm/kg)	29.5 ± 14.3
Knee extension strength/body weight non-affected side (Nm/kg)	40.5 ± 17.5
Six-minute walk test (m)	273.9 ± 13.1
Berg Balance Scale (score)	42.1 ± 15.2
Motor FIM at discharge (score)	71.1 ± 20.1

## Discussion

This study demonstrated that the direct effect of “Body function and structure” on “Home return” was not significant, while the indirect effect mediated by “Activity and participation” was significant. These findings indicate that solely improving physical and mental function is insufficient to promote discharge to home; such improvements must be integrated into activities of daily living (ADL) and participation. 

However, because this study was cross-sectional, causal inferences cannot be made. Future longitudinal or interventional research is needed to verify whether improvements in body function led to enhanced activity and participation, ultimately resulting in successful home return.

Prior research has consistently shown that enhanced ADL function is a strong predictor of discharge home after stroke, with higher ADL independence significantly increasing the likelihood of returning home [17-23]. Meta-analyses and large cohort studies have reported that ADL independence at admission is closely associated with discharge outcomes [18-23]. Additionally, higher scores on validated ADL assessments, such as the FIM and Barthel Index, are significant predictors of home discharge [18-23]. Early ADL performance, assessed within 48 hours post-stroke, has also been identified as a highly predictive factor using ADL-based decision tree models [22]. Pre-stroke ADL independence similarly predicts discharge destination [23].

From the caregivers' perspective, the degree of ADL impairment in patients with stroke is strongly associated with caregivers' psychological stress during rehabilitation. Caregivers of patients with severe ADL limitations report significantly higher anxiety and depression compared to controls. A pronounced negative correlation exists between patients' ADL scores and caregivers' psychological burden, indicating that as patients' independence declines, caregiver stress intensifies [24,25].

Caregivers providing ADL support often experience physical and emotional strain, frequently feeling overwhelmed and requiring additional assistance [25,26]. Declines in ADL function in patients with stroke substantially increase caregiver burden, diminish caregivers' quality of life, and elevate psychological distress [25,27]. Furthermore, patients' ADL impairment indirectly affects caregivers' mental health and well-being by increasing depression, anxiety, and stress [27,28]. A dynamic interaction exists between the emotional states of patients and caregivers; greater impairment or anxiety in patients is linked to higher depression and stress in caregivers [27].

From a clinical perspective, the present findings suggest that rehabilitation strategies should prioritize not only the recovery of body function but also the facilitation of activity and participation that bridge functional gains with home reintegration. Occupational therapists play a central role in translating improvements in physical performance into meaningful daily activities and participation outcomes. Strengthening this linkage may improve both patient independence and caregiver well-being.

Therefore, in planning home discharge, improving ADL is critical not only for patients but also for mitigating caregivers' psychological stress. Clinically, occupational therapists have a central role in translating gains in physical function into enhanced ADL. At discharge planning, it is essential for occupational therapists to adopt strategies that integrate improvements in body function and structure into “activity” and “participation.”

This study has several limitations. First, it employed a retrospective design, which may have led to inconsistencies in assessment and variations in the accuracy of medical records. Second, the sample size was relatively small (n = 67), potentially limiting the reliability of parameter estimates in structural equation modeling (SEM). Additionally, the indicators used to assess physical function and activity/participation were limited, excluding important factors such as cognitive function and social participation. Furthermore, the outcome of home return was treated as a simple binary variable, which may not fully capture the quality of post-discharge life or the level of support available. Lastly, potential confounding factors were not adequately controlled, requiring caution when interpreting causal relationships.

## Conclusions

In this study, mediation analysis demonstrated that body function and structure did not directly influence home return but exerted a significant indirect effect through activity and participation. These findings highlight the importance of enhancing not only physical function but also activity and participation to facilitate successful discharge planning. From a clinical perspective, occupational therapists play a key role in bridging physical improvements with activities of daily living and participation in real-life contexts. This underscores the need for interdisciplinary and client-centered approaches in rehabilitation practice to ensure a smoother transition to home life after stroke. This highlights that intervention strategies emphasizing activity and participation may more effectively support home return after stroke, even when functional recovery alone is insufficient.
